# Supplementary material for: Deletion of wfs1 Impairs Oligodendrocyte Precursor Cells Dorsal Distribution and Myelination Through the wfs1-hmgcs1 Axis in Zebrafish
Source: Biology (Basel). 2026 Aug 16;15(16):1402. doi: 10.3390/biology15161402 (PMC13510151; doi:10.3390/biology15161402)
Supplement: Supplementary file 1 [file biology-15-01402-s001.zip › File S1-Transcriptome sequencing data and differentially expressed genes (DEGs) in wfs1-- zebrafish at 5 dpf/2.KEGG/wfs1KOvsWT/DOWN/src/dre04145.html]

dre04145


- dre:57934

- Down regulated genes

57934(-1.25539807424297)

- dre:792699

- Down regulated genes

792699(-1.84389282152821)

- dre:792699

- Down regulated genes

792699(-1.84389282152821)

- dre:336681

- Down regulated genes

406811\_1(-1.09337424046752)

- dre:359840

- Down regulated genes

359840\_1(-1.77685900658053)

- dre:359840

- Down regulated genes

359840\_1(-1.77685900658053)

- dre:571584

- Down regulated genes

571584(-1.08054144451958)

- dre:403131

- Down regulated genes

403131(-4.42862381698358)

- dre:100007982

- Down regulated genes

100007982(-1.68529326567621)

- dre:100005358

- Down regulated genes

100005358(-3.40764150774661)

- dre:359840

- Down regulated genes

359840\_1(-1.77685900658053)

Close
